# Investigation into the Antihypertensive Effects of Diosmetin and Its Underlying Vascular Mechanisms Using Rat Model

**DOI:** 10.3390/ph15080951

**Published:** 2022-07-30

**Authors:** Taseer Ahmad, Adil Javed, Taous Khan, Yusuf S. Althobaiti, Aman Ullah, Farooq M. Almutairi, Abdul Jabbar Shah

**Affiliations:** 1 Department of Pharmacy, COMSATS University Islamabad, Abbottabad Campus, University Road, Abbottabad 22060, Pakistan; taseer.ahmad@uos.edu.pk (T.A.); adiljaved5@gmail.com (A.J.); taouskhan@cuiatd.edu.pk (T.K.); 2 Laboratory of Cardiovascular Research and Integrative Pharmacology, College of Pharmacy, University of Sargodha, Sargodha 40100, Pakistan; 3 Department of Pharmacology and Toxicology, College of Pharmacy, Taif University, Taif 21944, Saudi Arabia; ys.althobaiti@tu.edu.sa; 4 Addiction and Neuroscience Research Unit, Taif University, Taif 21944, Saudi Arabia; 5 College of Pharmaceutical Sciences, Shifa Tameer-e-Millat University, Islamabad 44000, Pakistan; amanullah767767@gmail.com; 6 Department of Clinical Laboratories Sciences, College of Applied Medical Sciences, University of Hafr Al-Batin, Hafr Al-Batin 39524, Saudi Arabia

**Keywords:** diosmetin, antihypertensive, vasorelaxant, antimuscarinic, calcium antagonist, K+ channel activation

## Abstract

Objective: Diosmetin is a flavonoid that is found in many important medicinal plants that have antihypertensive therapeutic potential. Diosmetin has been shown to have antiplatelet, anti-inflammatory and antioxidant properties, which suggests that it could be a potential candidate for use in antihypertensive therapy. Methods: In vivo and in vitro methods were used for our investigation into the antihypertensive effects of diosmetin. Results: Diosmetin significantly decreased the mean arterial pressure (MAP). The effects of diosmetin on the MAP and heart rate were more pronounced in hypertensive rats. To explore the involvement of the muscarinic receptors-linked NO pathway, Nω-nitro-L-arginine methyl ester (L-NAME) and atropine were pre-administered in vivo. The pretreatment with L-NAME did not significantly change the effects of diosmetin on the MAP by excluding the involvement of NO. Unlike L-NAME, the atropine pretreatment reduced the effects of diosmetin on the MAP, which demonstrated the role of the muscarinic receptors. In the in vitro study, diosmetin at lower concentrations produced endothelium-dependent and -independent (at higher concentrations) vasorelaxation, which was attenuated significantly by the presence of atropine and indomethacin but not L-NAME. Diosmetin was also tested for high K+-induced contractions. Diosmetin induced significant relaxation (similar to verapamil), which indicated its Ca2+ antagonistic effects. This was further confirmed by diosmetin shifting the CaCl_2_ CRCs toward the right due to its suppression of the maximum response. Diosmetin also suppressed phenylephrine peak formation, which indicated its antagonist effects on the release of Ca2+. Moreover, BaCl_2_ significantly inhibited the effects of diosmetin, followed by 4-AP and TEA, which suggested that the K+ channels had a role as well. Conclusions: The obtained data showed the Ca2+ channel antagonism, potassium channel activation and antimuscarinic receptor-linked vasodilatory effects of diosmetin, which demonstrated its antihypertensive potential.

## 1. Introduction

Flavonoids are phenolic compounds that are broadly distributed in medicinal plants, fruits and vegetables and are believed to have useful outcomes for human health. Studies have proven that the consumption of flavonoid-rich plants is linked to lower risks of cardiovascular diseases [1]. Flavonoids have also been reported to exert multiple biological effects, including antioxidant and anti-inflammatory effects, etc. [2,3].

**Figure 1:**
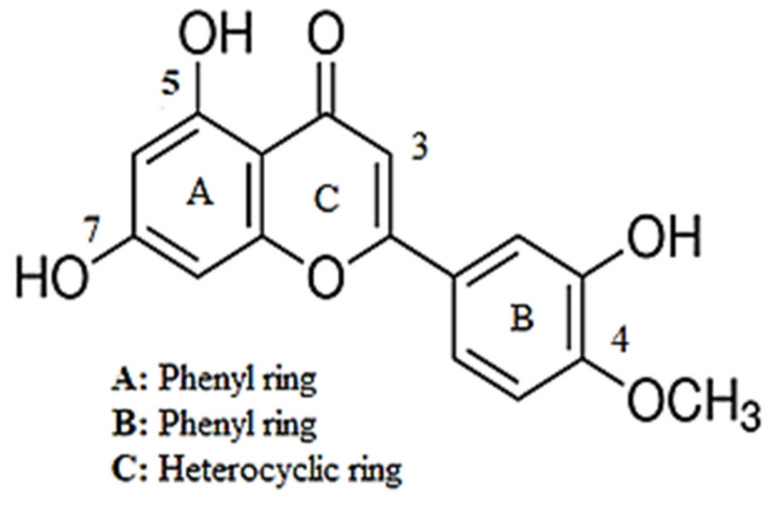
The chemical structure (5, 7, 3′-trihydroxy-4’-methoxyflavone) of diosmetin.

Diosmetin (5, 7, 3′-trihydroxy-4’-methoxyflavone; Figure 1) is a flavonoid that is found in citrus fruits and lemon peel. The diosmetin structure includes a 15-carbon skeleton, which consists of two phenyl rings (A and B) and a heterocyclic ring (C) [4,5,6]. *Citrus limon* L. (lemon) and other natural sources of diosmetin, such as *Rosmarinus officinalis* (rosemary) and *Olea europaea* (olive), have been shown to have therapeutic potential for the management of hypertension [7,8,9,10,11]. Approximately 27 mg/kg of diosmetin is present in the leaves of *Olea europaea* L., while *Citrus lemon* contains high levels of all flavonoids (80–95%), with 32 ± 4.95 mg/100 mL of diosmetin [6]. Moreover, olive leaf extract has been reported to reduce blood pressure [7] and dilate the aorta [12]. Furthermore, it has been reported that a flavanone derivative of diosmetin (hesperetin) causes smooth muscle relaxation via K^+^ channel activation and the release of NO [13]. As with other flavonoids, diosmetin has been reported as having anti-oxidant [14,15], hypolipidemic [16], anti-cancer [17], anti-microbial [18], anti-estrogenic [19], anti-inflammatory [15,20], anti-apoptotic [15] and anti-diabetic properties [21].

In addition, diosmetin also inhibits the smooth muscle contraction of the jejunum [13]. Diosmetin has also been reported to induce vasodilation in the porcine coronary artery [22]. However, the underlying mechanisms of diosmetin that cause this the smooth muscle relaxation still need to be investigated. Therefore, this study aimed to determine the vasorelaxant mechanisms and potential antihypertensive effects of diosmetin using rat models.

## 2. Results

### 2.1. Antihypertensive Activities of Diosmetin

#### Effects of Diosmetin on the MAP of SD Rats

**Figure 2:**
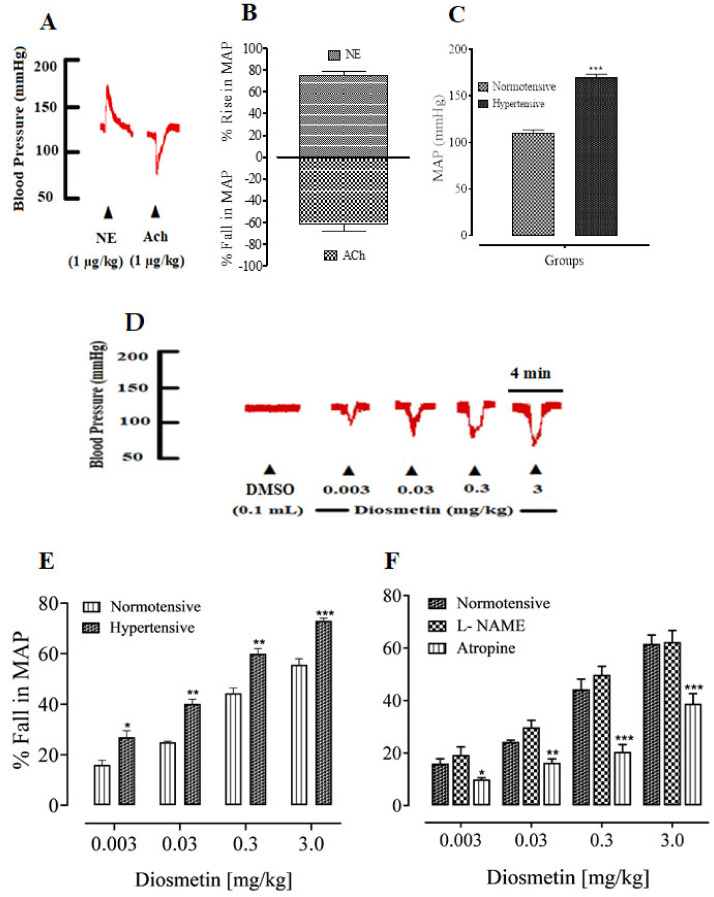
A representative tracing (**A**) showing the effects of norepinephrine (NE) and acetylcholine (Ach) on the mean arterial pressure (MAP): (**B**) the percentage increases and decreases in the BP of anesthetized normotensive rats; (**C**) the significant changes (*p* < 0.001) in the MAP of normotensive anesthetized rats; (**D**) A representative tracings of invasive blood pressure (BP) measurement tracings show the fall in blood pressure by the diosmetin in normotensive rats; (**E**) a comparison of the percentage decreases in the mean arterial pressure (MAP) of normotensive and hypertensive rats that were caused by diosmetin; (**F**) the effects of pretreatment with 20 mg/kg of L-NAME and 1 mg/kg of atropine in normotensive rats. Significant differences (*n* = 6): * *p* < 0.05; ** *p* < 0.01; *** *p* < 0.

When administered intravenously (IV), norepinephrine and acetylcholine produced a significant increase and decrease, respectively, in the MAP of both normotensive and hypertensive anesthetized rats (Figure 2A–C). After the validation of the protocol, diosmetin was administered via IV to normotensive and hypertensive anesthetized rats. The dose-dependent percentage decreases in MAP were 16.01 ± 0.77, 25.67 ± 1.33, 44.30 ± 2.20 and 56.0 ± 3.15 mmHg for doses of 0.003 to 3 mg/kg (as shown in Figure 2D). In hypertensive SD rats, diosmetin produced comparatively larger decreases in MAP, which were 27.78 ± 0.8, 40 ± 1.45, 60.33 ± 2.84 and 73.0 ± 2.52 mmHg (as shown in Figure 2E).

To further identify the mechanisms of the decrease in MAP, SD rats were treated with L-NAME. The decreases in MAP that were produced by diosmetin were 19.30 ± 1.85, 30.01 ± 1.70, 49.25 ± 1.10 and 62.33 ± 2.27 mmHg (Figure 2). Moreover, in rats that were pretreated with atropine, the percentage decreases in the MAP were 10.75 ± 1.29, 18.10 ± 2.30, 20.01 ± 2.50 and 39.50 ± 3.53 mmHg. In the presence of L-NAME, the changes in MAP were not significant, while the pretreatment with atropine significantly inhibited the decrease in MAP (Figure 2F). In addition, Table 1 shows the percentage decrease in heart rate (HR) after an injection of 0.003 to 3 mg/kg of diosmetin. The maximum decrease in heart rate (50%) was identified for the 3 mg/kg dose.

### 2.2. Vascular Reactivity

#### 2.2.1. Effects of Diosmetin on Aortic Tissues in Rats

**Figure 3:**
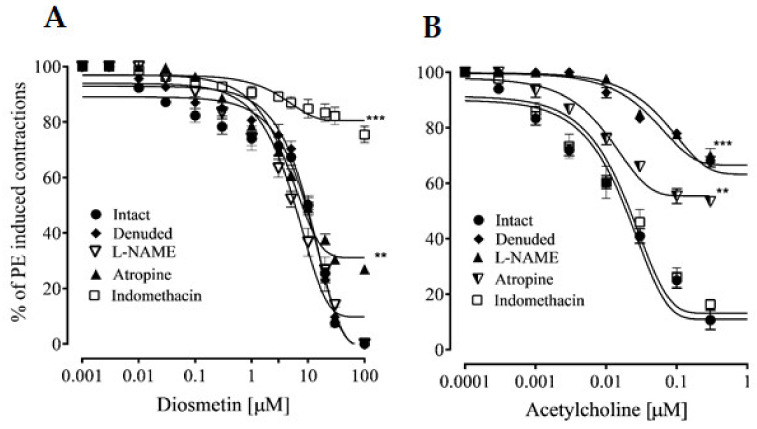
The effects of (**A**) diosmetin and (**B**) acetylcholine (ACh) on contractions that were induced by phenylephrine (PE; 1 µM) in intact and denuded aortic rings from rats that were pretreated with 10 µM of L-NAME, 1 µM of atropine and 1 µM of indomethacin. The relaxation responses are shown as means ± SEM (*n* = 6). Significant differences: ** *p* < 0.01; *** *p* < 0.001.

Intact aortic rings were pretreated with phenylephrine, followed by the cumulative addition of diosmetin. In response, diosmetin exhibited a vasorelaxant response with an EC_50_ value of 9.47 µg/mL (4.06–10.57). This vasorelaxation response was not significantly reduced in denuded tissues (Figure 3), with an EC_50_ value of 9.20 µg/mL (5.15–9.65), excluding the involvement of endothelium-dependent responses. In the presence of L-NAME (10 μM), the vasorelaxant response to phenylephrine-induced contractions was not significantly changed, with an EC50 value of 8.06 µg/mL (3.95–9.65) (Figure 3A). To explore the role of muscarinic receptors, intact aortic rings were pretreated with atropine (1 μM), which significantly reduced (27%) the vasorelaxant effects of diosmetin (Figure 3A). To further confirm the involvement of prostaglandins in the response that was produced by diosmetin, indomethacin was used to inhibit the cyclooxygenase pathways in the intact aortic rings. This pretreatment significantly inhibited (75%) the effects of diosmetin (Figure 3A). The effects of diosmetin were then compared to those of acetyl-choline (Figure 3B).

#### 2.2.2. Effects of Diosmetin on Ca^2+^ Signaling and Ca^2+^ Channels

**Figure 4:**
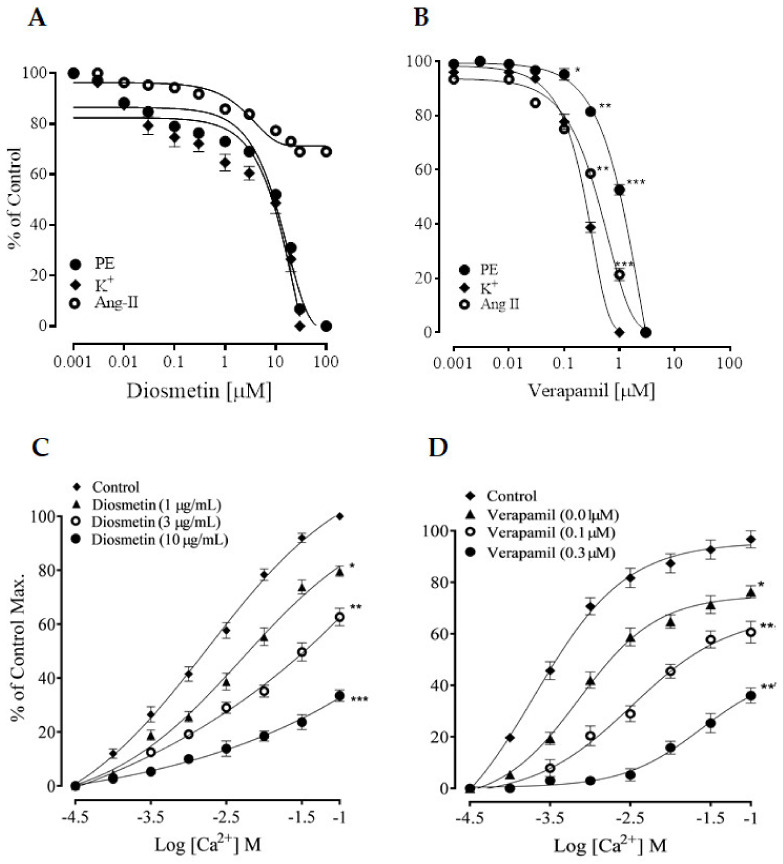
(**A**,**B**)The vasorelaxant responses of diosmetin and verapamil to phenylephrine (PE), high K^+^ and 5 µM of Ang II; (**C**,**D**) the effects of diosmetin and verapamil on the calcium concentration response curves (CRCs) in a calcium-free medium using the aortic rings of rats. The relaxation and contractile responses are shown as means ± SEM (*n* = 6). Significant differences vs. control: * *p* < 0.05; ** *p* < 0.01; *** *p* < 0.001.

Diosmetin produced a concentration-dependent vasorelaxation in response to phenylephrine and K+ (80 mM) in the pretreated aortic rings, with EC_50_ values of 10.06 (6.90–11.87) and 10.13 μg/mL (5.50–10.20). These effects of diosmetin were similar to those of verapamil (Figure 4A,B). Unlike verapamil, diosmetin only induced 20% relaxation in response to Ang II (5 uM) (as shown in Figure 4A,B).

Moreover, the different concentrations (1–10 µg/mL) of diosmetin significantly (*p* < 0.001) affected the CaCl_2_ (0.01–1 mM) CRCs in a calcium-free medium and shifted the CRCs that were produced by CaCl_2_ toward the right (Figure 4C), as with verapamil (Figure 4D).

### 2.3. Effects of Diosmetin on Intracellular Calcium Stores

**Figure 5:**
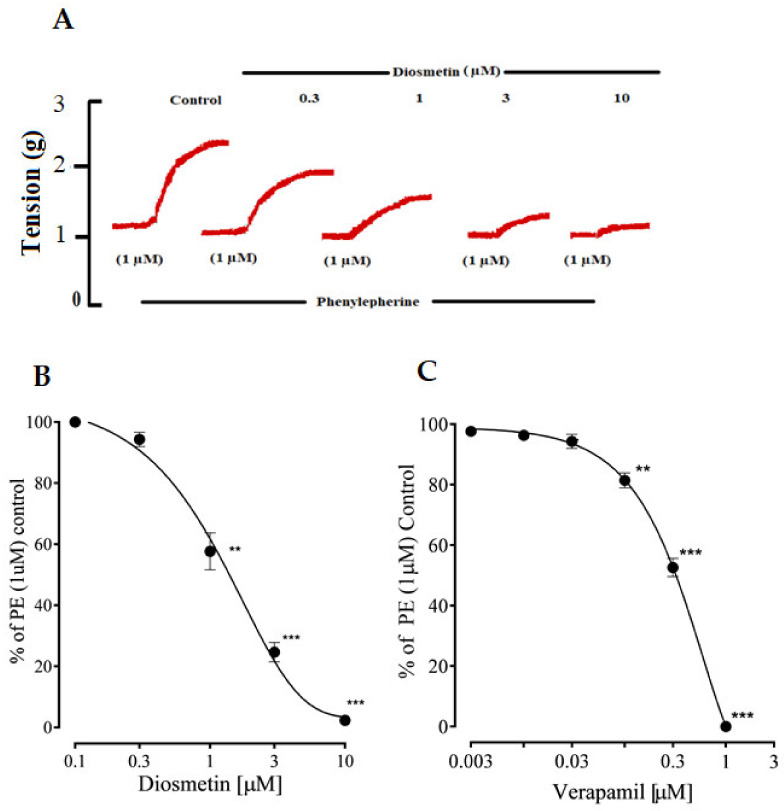
A representative tracing of (**A**) phenylephrine, which inhibited the presence of increasing concentrations of the diosmetin in the calcium-free medium; (**B**,**C**) the increasing concentrations of diosmetin and verapamil, respectively, which significantly inhibited phenylephrine (PE) peak formation in the calcium-free medium. Significant differences vs. control (*n* = 6): ** *p* < 0.01; *** *p* < 0.001.

The pretreatment of the isolated aortic rings with different concentrations (0.3–10 µg/mL) of diosmetin reduced the phenylephrine transient contractile responses in a calcium-free Krebs solution. The effects of diosmetin were then compared to those of verapamil (Figure 5A–C).

### 2.4. Effects of Diosmetin on Aortic Tissues of Rats Pretreated with K^+^ Channel Inhibitors

**Figure 6:**
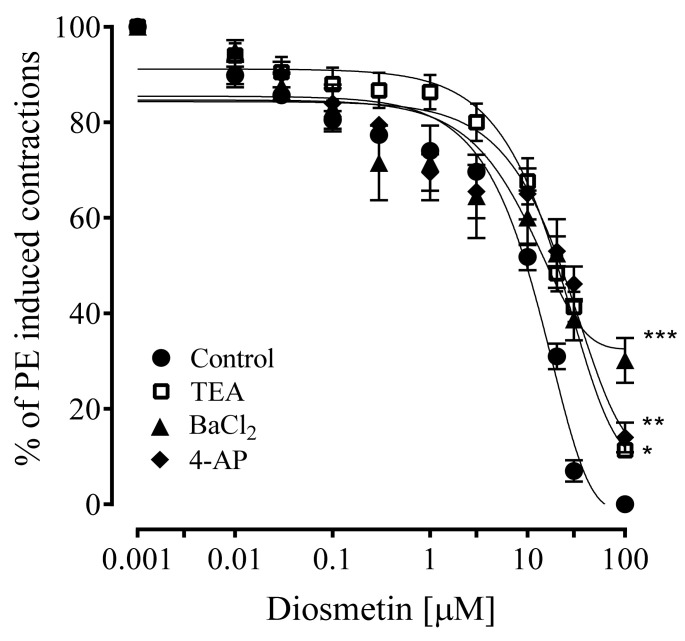
The response of diosmetin to phenylephrine (1 µM) in the control and in the aortic rings of rats that were pretreated with tetraethylammonium (TEA; 5 mM), barium chloride (BaCl_2_; 30 µM) and 4-aminopyridine (4-AP; 1 mM). Significant differences vs. the control (*n* = 6): * *p* < 0.05; ** *p* < 0.01; *** *p* < 0.001.

To explore the involvement of K^+^ channels in the vasorelaxant effects of diosmetin, potassium channel activation and various K^+^ channel blockers were investigated. The vasorelaxant effects of diosmetin were significantly (* *p* < 0.05, ** *p* < 0.01 and *** *p* < 0.001) attenuated by pretreatment with TEA (11%), BaCl_2_ (30%) and 4-AP (14%) versus the control, with EC_50_ values of 10.50 (4.29–8.74), 15.70 (8.50–15.40) and 19.80 for TEA, BaCl_2_ and 4-AP, respectively (Figure 6).

## 3. Discussion

Phytochemicals are abundant in plants and have widely recognized pharmacological actions, including cardiovascular effects [23]. The present study revealed the antihypertensive effects of the flavonoid diosmetin in both normotensive and hypertensive SD rats. Diosmetin caused a dose-dependent decrease in the MAP of anesthetized normotensive rats. However, further confirmation was needed for its effects on hypertensive rats; therefore, diosmetin was also tested in an 8% salt-induced hypertensive rat model. Intravenous injections of diosmetin were administered to hypertensive rats, which also induced decreases in the MAP that were comparatively more significant (*p* < 0.001), thus indicating that diosmetin could be a promising antihypertensive agent.

This decrease in MAP that was caused by diosmetin could be the outcome of its interactions with various endogenous mediators that act through various receptors or channels. To explore the role of muscarinic receptors in the antihypertensive effects of diosmetin, atropine (a muscarinic receptor antagonist) was pre-administered [24]. This pretreatment significantly reduced (23%) the blood pressure lowering effects of diosmetin, which suggested that the muscarinic receptor stimulation that was caused by diosmetin played a role in its antihypertensive effects. in the vessels in the muscarinic receptors and the NO pathway are linked [25]. To investigate the role of this pathway in the antihypertensive effects of diosmetin, normotensive rats were pretreated with L-NAME, which is a nitric oxide synthase inhibitor [26]. This pretreatment did not change the responses of diosmetin, which indicated that NO was not the endogenous vascular mediator.

Furthermore, according to the antihypertensive in vivo data, diosmetin induced significant decreases (50%) in heart rate. At this stage, it was not clear whether diosmetin activated the cardiac muscarinic receptors; however, the decrease in heart rate suggested that it also activated cardiac receptors. Additional in vitro studies were carried out on the aortas of rats to examine the antihypertensive effects of diosmetin.

Endothelial cells release NO, which is an important regulator of some vascular functions [27,28]. In addition, different channels can lead to vasorelaxation, such as the blockage of the Ca^2+^ channels and the activation of K^+^ channels in the smooth muscle [29]. To explore the effects of diosmetin on endothelial and smooth muscle cells, the aortas of normotensive rats was tested. Vasoconstrictors, such as phenylephrine, high K^+^ and Ang II, were used to initially confirm the different vascular effects of diosmetin. Diosmetin induced vasorelaxation in the induced contractions, which initially confirmed its calcium-dependent signaling pathways.

The vasorelaxation that was caused by diosmetin was not significantly inhibited with the removal of endothelium, which indicated that vascular endothelium or mediators that were derived from endothelium were not involved. Further experiments supported this finding using the pretreatment of aortic rings with L-NAME, which did not modify the relaxant effects of diosmetin. Based on the observations from the in vivo experiments in which atropine pretreatment partly attenuated the antihypertensive effects of diosmetin, the aortic rings were also pretreated with atropine to investigate the role of the vascular muscarinic receptors. Our findings indicated that this pretreatment with atropine modified (27%) the vasorelaxant effects of diosmetin, which indicated that the vascular muscarinic receptors were partly involved in the vasorelaxant effects of diosmetin. This confirmed that the vascular muscarinic receptors mediated the antihypertensive effects of diosmetin. However, further experiments are required to examine the mechanism(s) of the vasorelaxant effects of diosmetin.

In addition to NO, endothelial cells also produce prostacyclin [30]; so, the intact aortic rings were pretreated with indomethacin, which is a prostaglandin inhibitor [31]. Interestingly, the pretreatment with indomethacin significantly inhibited (75%) the vasorelaxant effects of diosmetin. These data indicated that the vasorelaxant effects of diosmetin were predominately mediated by vasodilatory prostaglandin, which was most probably prostacyclin. However, if diosmetin induced endothelium-independent effects, the question then arose of how its effects were reversed with indomethacin because prostacyclin is also synthesized in endothelium cells. Previous studies have shown that indomethacin is also a potassium channel modulator, which decreases the expression of voltage-gated potassium (Kv) channels [32] and inhibits ATP-sensitive potassium (K_ATP_) channels [33,34]. Moreover, indomethacin has also been reported to have a role in the regulation of Ca^2+^ influx and calcium release from stores [34,35]. Our results showed that diosmetin activated potassium channels and inhibited intracellular Ca^2+^ release. So, reduced diosmetin responses could be due to the downregulation of potassium channels and the increase in Ca^2+^ concentration that were caused by indomethacin.

As seen before, diosmetin reduced phenylephrine-induced contractions, which demonstrated its promising inhibitory response to the intracellular release of Ca^2+^. Phenylephrine has been reported to induce aortic contractions by binding to G_q_ protein-coupled α1-adrenergic receptors, which leads to the generation of the IP_3_ and DAG pathways. DAG triggers the protein kinase C (PKC). The IP_3_ binds to its receptors (IP_3_R) in the sarcoplasmic reticulum (SR) and releases Ca^2+^ to produce contractions. The nature of phenylephrine contractions is biphasic, i.e., a fast phase that is followed by a slow phase [36,37]. In the fast phase, the contractions are due to the release of Ca^2+^ from stores, while in the slow phase, they are dependent on the influx of calcium from ROCs [38,39]. The inhibitory effects of diosmetin were observed on the Ca^2+^ release from intracellular Ca^2+^ stores, as with a standard Ca^2+^ influx inhibitor (e.g., verapamil) [40]. So, it appeared as though diosmetin had inhibitory effects on the release of IP_3_-dependent Ca^2+^ from the sarcoplasmic reticulum (SR). These findings encouraged us to conduct further experiments to study whether diosmetin also had an effect on Ca^2+^ channels.

High K^+^ was used to pretreat the aortic rings of the rats, then diosmetin was added cumulatively to inhibit the precontractions in a concentration-dependent manner, similar to verapamil. The contractions that were produced by the high K^+^ depended on the release of Ca^2+^ through VDCs [41,42]. It has been concluded that that drugs that prevent contractions that are induced by high K+ can be considered as Ca^2+^ channel blockers [43]. So, it could be suggested that diosmetin could also prevent Ca^2+^ influx through VDCs, which was further studied by producing CaCl_2_ CRCs in a calcium-free medium. The rightward shifts in the CRCs showed that diosmetin also antagonized calcium influx through VDCs, which was similar to verapamil.

We conducted an experiment to see whether diosmetin had effects on other mediators that increase vascular resistance, such as angiotensin II (Ang II), as rat aortas contain Ang II receptors [44]. Isolated aortic rings from the rats were pretreated with Ang II and then diosmetin was added cumulatively, which induced a gradual inhibition of the induced contractions, reaching a maximum of about 20%. This suggested that diosmetin had minimal inhibitor effects on Ang II receptors (unlike verapamil, which inhibits Ang II precontractions), thus indicating that diosmetin was a different Ca^2+^ channel blocker from verapamil.

In comparison to verapamil, diosmetin induced vasorelaxation against high K^+^ precontractions that was > 10 times higher, which indicated that it could act on some other vascular channels. One such possibility could be the stimulation of potassium channels because vasodilators that are dependent on K^+^ channel activation lose their effects when exposed to high K^+^. High K^+^ reduces the K^+^ concentration gradient in the plasma membrane, which leads to the ineffective activation of K^+^ channels [45]. So, high K+ leads to the inhibition of K^+^ channels [46].

The activation of K^+^ channels plays a key role in vascular tone regulation. Different types of K^+^ channels are present in smooth muscle, such as K^+^ voltage-gated channels (Kv), calcium-activated K^+^ channels (K_Ca_) and inward rectifying K+ channels (K_ir_). To confirm the role of K^+^ channels in the vasorelaxant responses that are produced by diosmetin, the different K^+^ channel inhibitors were pretreated. Aortic rings that were pretreated with TEA (K_Ca_ channel blocker) [47], barium chloride (K_ir_ channel inhibitor) [48] and 4-AP (K_v_ channel blocker) [49] demonstrated significantly (*p* < 0.05, *p* < 0.01 and *p* < 0.001) reduced vasorelaxant effects of diosmetin. The percentage decrease was more significant in the presence of BaCl_2_ (30%) and 4-AP (14%) compared to TEA (11%). These interesting outcomes indicated that potassium channel stimulation partly contributed to the vasorelaxant effects of diosmetin.

## 4. Methodology

### 4.1. Drugs and Reagents

The acetylcholine chloride, phenylephrine hydrochloride, atropine sulfate, potassium chloride, L-NAME, atropine, indomethacin, verapamil hydrochloride, 4-aminopyridine (4-AP), tetraethylammonium chloride (TEA), barium chloride (BaCl_2_), angiotensin II (Ang II) and dimethyl sulfoxide (DMSO) were purchased from Sigma-Aldrich, St. Louis, MO, USA. The EGTA was imported from Alfa Aesar, Heysham, UK. The heparin and thiopental vials were provided by Abbot Lab., Karachi, Pakistan. The diosmetin was purchased from Tocris Bioscience (Bristol, UK). All drugs were dissolved in distilled water/normal saline (except for indometha cin and ionomycin, which were dissolved in absolute ethanol). The tested diosmetin compound was first dissolved in DMSO (dimethyl sulfoxide) and then diluted with distilled water (the final concentration for the in vitro studies was <0.1% DMSO and the in vivo studies contained ≤ 1% DMSO).

### 4.2. Experimental Rats

Adult Sprague–Dawley (SD) rats, aged 8–10 weeks, were used to perform the antihypertensive and vascular reactivity studies. The NRC guidelines were followed for all experimental protocols [50], which were approved by the EC of the Department of Pharmacy, CUI, Abbottabad campus, in a meeting that was held on 18 May 2013 (vide notification: EC/PHM/05-2013/CUI/ATD).

### 4.3. MAP Measurement in Normotensive SD Rats

Anesthesia was induced in the SD rats using an intraperitoneal (IP) injection of pentothal (60 mg/kg). The trachea was exposed by a small mid-tracheal incision (approximately 1 cm). Polyethylene (PE-20) tubes were used for the tracheal intubation. Moreover, PE-50 tubes were used to cannulate the carotid artery (to inject heparin) and the right jugular vein (to administer heparin, normal saline, diosmetin and standard drugs). The blood pressure was recorded using invasive BP apparatus [51].

After half an hour of equilibration, 1 µg/kg of norepinephrine and 1 µg/kg of acetylcholine were administered to confirm the hypertensive and hypotensive responses in the rats, respectively. Normal saline (0.1 mL) was injected a few times during the experiments. Diosmetin was injected at different doses to examine the significant dose range. The involvement of the nitric oxide pathway and the muscarinic receptors was investigated using SD rats that were pretreated with 20 mg/kg of L-NAME and 1 mg/kg of atropine in separate experiments. The L-NAME dose was followed by the atropine when the MAP returned to a normal level. The changes in the MAP were calculated using the following formula with the SBP and DBP values [52,53]:

MAP = SBP + 2 (DBP)/3

The following formula was used to calculate the percentage decrease in MAP:

$$\frac{\mathrm{Control}-\mathrm{Fall}}{\mathrm{Control}}\times 100\%$$

### 4.4. MAP Measurement in Hypertensive SD Rats

The rats were divided in to two groups, each containing six animals. Group 1 was considered as the control and only received normal saline, while group 2 received an 8% NaCl-rich diet for 14 days. SD rats with systolic BP rates of more than 140 mmHg and diastolic BP rates of more than 90 mmHg were considered hypertensive. The rats were all fed with a normal diet and water in the 24 h prior to the experiments. Subsequently, the in vivo blood pressure of the rats was measured as previously described [52,53,54].

### 4.5. Preparation of Isolated Aortic Rings of Rats

The thoracic aorta was cautiously removed from the SD rats to avoid endothelium damage. Then, 2-mm aortic rings were prepared. The aortic rings were hung in baths that were filled with Krebs solution and were constantly bubbled with carbogen gas at 37 °C. Then, 2 g of tension was applied and equilibrated for 30–45 min. The isometric tension changes were noted using a tissue organ bath that was connected to PowerLab [55].

### 4.6. The Effects of Diosmetin on Precontractions Induced by Standard Vasoconstrictors

Steady-state contractions were achieved using standard vasoconstrictor drugs, such as 1 µM of phenylephrine, K^+^ (80 mM) and 5 µM of Ang II, in the aortic preparations. Diosmetin was added cumulatively to examine its response to the contractions that were induced by the above-mentioned standard drugs. In some aortic rings, the intimal surface was gently rubbed with forceps to deliberately damage the endothelium and were considered denuded when no significant relaxation (< 80%) was observed after the addition of acetylcholine (0.1 µM) [54,56].

### 4.7. Diosmetin Responses in the Presence of Different Vascular Pathway Inhibitors

The endothelium-intact aortic rings were pretreated for 20 min with 10 µM of L-NAME, 1 µM of atropine and 1 µM of indomethacin. The responses of diosmetin in the absence and presence of the above-mentioned inhibitors of different vascular pathways were then compared [57,58].

### 4.8. Effects of Diosmetin on Ca^2+^ Channels

First, the tissues were stabilized and then the integrity of the tissues was confirmed using the precontractions that were induced by K^+^ (80 mM). After the stabilization of tissues, CRCs were recorded for 0.01–10.0 mM of CaCl_2_ with the simultaneous replacement of the normal Krebs solution with 0.2 mM of EGTA, which contained Ca^2+^-free Krebs solution. To investigate the calcium antagonistic response of diosmetin, some isolated aortic rings were pretreated with diosmetin (μg/mL) and the contractions were analyzed for the individual concentrations of CaCl_2_ (0.01–10.0 mM). Some additional aortic rings were hung in tissue organ baths for 10–15 min and were pretreated with verapamil for the construction of CaCl_2_ CRCs to confirm the possible calcium antagonistic effects [40,54,58].

### 4.9. Effects of Diosmetin on Intracellular Ca^2+^ Stores

To see whether diosmetin had any effects on intracellular Ca^2+^ movement, the aortic rings were equilibrated in normal Krebs solution. The primary phenylephrine peak in normal solution was followed by the subsequent phenylephrine peak in the calcium-free solution. The aortic rings were pretreated with diosmetin in different concentrations (0.003–10 μg/mL) in an organ bath for 30 min prior to phenylephrine administration. For comparison, the same protocol was also run with verapamil [51,54,59].

### 4.10. Effects of Diosmetin on K^+^ Channel Inhibitors

The aortic rings were exposed to phenylephrine to induce contractions in the absence and presence of potassium channel inhibitors (tetraethylammonium (5 mM) [60], 1 mM of 4-aminopyridine [61] and 30 µM of barium chloride [62]) in different experiments. The potassium channel inhibitors were added 20 min prior to the administration of phenylephrine. After stable contractions were induced by phenylephrine, diosmetin was added to the bath cumulatively.

## 5. Conclusions

This study confirmed that diosmetin is an important flavonoid that could be a potential candidate for antihypertensive therapy. The effects of diosmetin are mainly due to the vasorelaxation that is caused by the blockage of the Ca^+2^ channel, K^+^ channel activation and the indomethacin-sensitive vasodilator prostaglandin. To better our understanding of these findings, further electrophysiological studies should be performed.

## Figures and Tables

**Table 1:** The percentage decrease in the BP and HR of anesthetized rats after the injection of different doses of diosmetin.

Dose (mg/kg)	Diosmetin	
	BP (%)	HR (%)
Control	99.8 ± 0.07	99.5 ± 0.10
0.003	16 ± 2.47 *	13 ± 1.90 *
0.03	24 ± 1.77 *	28 ± 2.50 *
0.3	54 ± 2.03 ***	38 ± 3.05 **
3	61 ± 1.03 ***	50 ± 2.04 ***

Significant differences (*n* = 6): * *p* < 0.05; ** *p* < 0.01; *** *p* < 0.001.

## Data Availability

Data is contained within the article.

## References

[1] Wang X., Ouyang Y.Y., Liu J., Zhao G. (2014). Flavonoid intake and risk of CVD: A systematic review and meta-analysis of prospective cohort studies. Br. J. Nutr..

[2] Ahmed S.I., Hayat M.Q., Tahir M., Mansoor Q., Ismail M., Keck K., Bates R.B. (2016). Pharmacologically active flavonoids from the anticancer; Antioxidant and antimicrobial extracts of *Cassia angustifolia* Vahl. BMC Complement. Altern Med..

[3] Meng X.H., Liu C., Fan R., Zhu L.F., Yang S.X., Zhu H.T., Wang D., Yang C.R., Zhang Y.J. (2018). Antioxidative Flavan-3-ol Dimers from the Leaves of *Camellia fangchengensis*. J. Agric. Food Chem..

[4] Miyake Y., Mochizuki M., Okada M., Hiramitsu M., Morimitsu Y., Osawa T. (2007). Isolation of Antioxidative Phenolic Glucosides from Lemon Juice and Their Suppressive Effect on the Expression of Blood Adhesion Molecules. Biosci. Biotechnol. Biochem..

[5] Horowitz R. (1956). Flavonoids of Citrus. I. Isolation of Diosmin from Lemons (*Citrus Limon*). J. Org. Chem. Res..

[6] Caristi C., Bellocco E., Gargiulli C., Toscano G., Leuzzi U. (2006). Flavone-Di-C-Glycosides in Citrus Juices from Southern Italy. Food Chem..

[7] Khayyal M.T., El-Ghazaly M.A., Abdallah D.M., Nassar N.N., Okpanyi S.N., Kreuter M.H. (2002). Blood pressure lowering effect of an olive leaf extract (*Olea europaed*) in L-NAME induced hypertension in rats. Arzneimittelforschung.

[8] Meirinhos J., Silva B.M., Valentao P., Seabra R.M., Pereira J.A., Dias A., Andrade P.B., Ferreres F. (2005). Analysis and quantification of flavonoidic compounds from Portuguese olive (*Olea europaea L*.) leaf cultivars. Nat. Prod. Res..

[9] Kato Y., Domoto T., Hiramitsu M., Katagiri T., Sato K., Miyake Y., Aoi S., Ishihara K., Ikeda H., Umei N. (2014). Effect on blood pressure of daily lemon ingestion and walking. J. Nutr. Metab..

[10] Avello M., Jofre P., Pastene E., Fernandez P. (2014). Use of *Citrus Limon,* L. (lemon) in Treating Blood Pressure Sudden Rises. Int. J. Pharmacog. Phytochem. Res..

[11] Raskovic A., Milanovic I., Pavlovic N., Cebovic T., Vukmirovic S., Mikov M. (2014). Antioxidant activity of rosemary (*Rosmarinus officinalis* L.) essential oil and its hepatoprotective potential. BMC Complement. Altern. Med..

[12] Zarzuelo A., Duarte J., Jimenez J., Gonzalez M., Utrilla M.P. (1991). Vasodilator effect of olive leaf. Planta Med..

[13] Mendel M., Chłopecka M., Dziekan N., Karlik W. (2016). Antispasmodic effect of selected Citrus flavonoids on rat isolated jejunum specimens. Eur. J. Pharmacol..

[14] Liao W., Ning Z., Chen L., Wei Q., Yuan E., Yang J., Ren J. (2014). Intracellular antioxidant detoxifying effects of diosmetin on 2.; 2-azobis (2-amidinopropane) dihydrochloride (AAPH)-induced oxidative stress through inhibition of reactive oxygen species generation. J. Agric. Food Chem..

[15] Yang Y., Gong X.B., Huang L.G., Wang Z.X., Wan R.Z., Zhang P., Zhang Q.Y., Chen Z., Zhang B.S. (2017). Diosmetin exerts anti-oxidative; Anti-inflammatory and anti-apoptotic effects to protect against endotoxin-induced acute hepatic failure in mice. Oncotarget.

[16] Andreeva O.A., Ivashev M.N., Ozimina I.I., Maslikova G.V. (1998). Diosmetin Glycosides from Caucasian Vetch: Isolation and Study of Biological Activity. Pharm. Chem. J..

[17] Wang P., Gao C., Wang W., Yao L.P., Zhang J., Zhang S.D., Li J., Fang S.H., Fu Y.J. (2018). Juglone induces apoptosis and autophagy via modulation of mitogen-activated protein kinase pathways in human hepatocellular carcinoma cells. Food Chem. Toxicol..

[18] Meng J.C., Zhu Q.X., Tan R.X. (2000). New antimicrobial monoand sesquiterpenes from *Soroseris hookeriana* subsp.erysimoides. Planta Med..

[19] Yoshikawa M., Uemura T., Shimoda H., Kishi A., Kawahara Y., Matsuda H. (2000). Medicinal foodstuffs. XVIII. Phytoestrogens from the aerial part of *Petroselinum crispum* MIll. (Parsley) and structures of 6″-acetylapiin and a new monoterpene glycoside, petroside. Chem. Pharm. Bull..

[20] Ge A., Liu Y., Zeng X., Kong H., Ma Y., Zhang J., Bai F., Huang M. (2015). Effect of diosmetin on airway remodeling in a murine model of chronic asthma. Acta Biochim. Biophys. Sin..

[21] Juarez-Reyes K., Brindis F., Medina-Campos O.N., Pedraza-Chaverri J., Bye R., Linares E., Mata R. (2015). Hypoglycemic, antihyperglycemic, and antioxidant effects of the edible plant *Anoda cristata*. J. Ethnopharmacol..

[22] Ahmad T., Shah A.J., Khan T., Roberts R. (2020). Mechanism underlying the vasodilation induced by diosmetin in porcine coronary artery. Eur. J..

[23] Vasanthi H.R., ShriShriMal N., Das D.K. (2012). Phytochemicals from Plants to Combat Cardiovascular Disease. Curr. Med. Chem..

[24] Arunlakhshana O., Schild H.O. (1959). Some quantitative uses of drug antagonists. Brit. J. Pharmacol..

[25] Yao Z., Gross G.J. (1993). Role of nitric oxide; Muscarinic receptors; And the ATP-sensitive K^+^ channel in mediating the effects of acetylcholine to mimic preconditioning in dogs. Circ. Res..

[26] Pfeiffer S., Leopold E., Schmidt K., Brunner F., Mayer B. (1996). Inhibition of nitric oxide synthesis by NG-nitro-L-arginine methyl ester (L-NAME): Requirement for bioactivation to the free acid, NG-nitro-L-arginine. Br. J. Pharmacol..

[27] Kruger-Genge A., Blocki A., Franke R.P., Jung F. (2019). Vascular Endothelial Cell Biology: An Update. Int. J. Mol. Sci..

[28] Lo Y.C., Tsou H.H., Lin R.J., Wu D.C., Wu B.N., Lin Y.T., Chen J. (2005). Endothelium-dependent and-independent vasorelaxation by a theophylline derivative MCPT: Roles of cyclic nucleotides, potassium channel opening and phosphodiesterase inhibition. Life Sci..

[29] Onsa-ard A., Shimbhu D., Tocharus J., Sutheerawattananonda M., Pantan R., Tocharus C. (2013). Hypotensive and vasorelaxant effects of sericin-derived oligopeptides in rats. ISRN Pharmacol..

[30] Prins B.A., Hu R.M., Nazario B., Pedram A., Frank H.J., Weber M.A., Levin E.R. (1994). Prostaglandin E2 and prostacyclin inhibit the production and secretion of endothelin from cultured endothelial cells. J. Biol. Chem..

[31] Roghani-Dehkordi F., Roghani M. (2016). The vasorelaxant effect of simvastatin in isolated aorta from diabetic rats. ARYA Atheroscler..

[32] Silver K., Littlejohn A., Thomas L., Marsh E., Lillich J.D. (2015). Inhibition of Kv channel expression by NSAIDs depolarizes membrane potential and inhibits cell migration by disrupting calpain signaling. Biochem. Pharmacol..

[33] Toroudi H.P., Rahgozar M., Bakhtiarian A., Djahanguiri B. (1999). Potassium channel modulators and indomethacin-induced gastric ulceration in rats. Scand. J. Gastroenterol..

[34] Menozzi A., Pozzoli C., Poli E., Passeri B., Gianelli P., Bertini S. (2011). Diazoxide attenuates indomethacin-induced small intestinal damage in the rat. Eur. J. Pharmacol..

[35] Hu H., Tian J., Zhu Y., Wang C., Xiao R., Herz J.M., Wood J.D., Zhu M.X. (2010). Activation of TRPA1 channels by fenamate nonsteroidal anti-inflammatory drugs. Pflug. Arch. Eur. J. Phy..

[36] Earley S., Brayden J.E. (2015). Transient Receptor Potential Channels in the Vasculature. Physiol. Rev..

[37] Bohr D.F. (1963). Vascular smooth muscle: Dual effects of calcium. Science.

[38] Yen M.H., Wu C.C., Chiou W.F. (1988). Partially endothelium-dependent vasodilator effect of adenosine in rat aorta. Hypertension.

[39] Karaki H., Ozaki H., Hori M., Mitsui-Saito M., Amano K., Harada K., Miyamoto S., Nakazawa H., Won K.J., Sato K. (1997). Calcium movements, distribution, and functions in smooth muscle. Pharmacol. Rev..

[40] Chan S.S.K., Choi A.O.K., Jones R.L., Lin G. (2006). Mechanisms underlying the vasorelaxing effects of butylidenephthalide, an active constituent of Ligusticum chuanxiong, in rat isolated aorta. Eur. J. Pharmacol..

[41] Zhang Y., Hermanson M.E., Eddinger T.J. (2013). Tonic and Phasic Smooth Muscle Contraction Is Not Regulated by the PKCα—CPI-17 Pathway in Swine Stomach Antrum and Fundus. PLoS ONE.

[42] Bolton T.B. (1979). Mechanisms of action of transmitters and other substances on smooth muscle. Physiol. Rev..

[43] Wu X.L., Wang Y.Y., Cheng J., Zhao Y.Y. (2006). Calcium channel blocking activity of calycosin, a major active component of *Astragali Radix*, on rat aorta. Acta Pharmacol. Sin..

[44] Chen H.C., Bouchie J.L., Perez A.S., Clermont A.C., Izumo S., Hampe J., Feener E.P. (2000). Role of the angiotensin AT1 receptor in rat aortic and cardiac PAI-1 gene expression. Arterioscler. Thromb. Vasc. Biol..

[45] Adeagbo A., Triggle C. (1993). Varying extracellular [K^+^]: A functional approach to separating EDHF- and EDNO-related mechanisms in perfused rat mesenteric arterial bed. J. Cardiovasc. Pharmacol..

[46] Ross G.R., Yallampalli C. (2006). Endothelium-independent relaxation by adrenomedullin in pregnant rat mesenteric artery: Role of cAMP-dependent protein kinase A and calcium-activated potassium channels. J. Pharmacol. Exp. Ther..

[47] Iozzi D., Schubert R., Kalenchuk V.U., Neri A., Sgaragli G., Fusi F., Saponara S. (2013). Quercetin relaxes rat tail main artery partly via a PKG-mediated stimulation of KCa 1.1 channels. Acta Physiol..

[48] Zhu X.M., Fang L.H., Li Y.J., Du G.H. (2007). Endothelium-dependent and -independent relaxation induced by pinocembrin in rat aortic rings. Vascul. Pharmacol..

[49] Novakovic A., Bukarica L.G., Kanjuh V., Heinle H. (2006). Potassium channels-mediated vasorelaxation of rat aorta induced by resveratrol. Basic Clin. Pharmacol. Toxicol..

[50] National Research Council (NRC) (1996). Guide for the Care and Use of Laboratory Animals.

[51] Shah A.J., Gilani A.H. (2009). Blood pressure lowering and vascular modulator effects of *Acorus calamus* extract are mediated through multiple pathways. Cardiovasc. Pharmacol..

[52] Parasuraman S., Raveendran R. (2012). Measurement of invasive blood pressure in rats. J. Pharmacol. Pharmacother..

[53] Van Vliet B.N., Montani J.P. (2008). The time course of salt-induced hypertension, and why it matters. Int. J. Obes..

[54] Qayyum R., Qamar H.M.D., Khan S., Salma U., Khan T., Shah A.J. (2016). Mechanisms underlying the antihypertensive properties of *Urtica dioica*. J. Transl. Med..

[55] Furchgott R.F., Zawadzki J.V. (1980). The obligatory role of endothelial cells in the relaxation of arterial smooth muscle by acetylcholine. Nature.

[56] Faraci F.M., Heistad D.D. (1998). Regulation of the Cerebral Circulation: Role of Endothelium and Potassium Channels. Physiol. Rev..

[57] Yam M.F., Tan C.S., Ahmad M., Ruan S. (2016). Vasorelaxant action of the chloroform fraction of *Orthosiphon stamineus* via NO/cGMP pathway, potassium and calcium channels. Am. J. Chin. Med..

[58] Senejoux F., Girard C., Kerram P., Aisa H.A., Berthelot A., Bévalot F., Demougeot C. (2010). Mechanisms of vasorelaxation induced by *Ziziphora clinopodioides* Lam. (Lamiaceae) extract in rat thoracic aorta. J. Ethnopharmacol..

[59] Sonkusare S., Palade P.T., Marsh J.D., Telemaque S., Pesic A., Rusch N.J. (2006). Vascular calcium channels and high blood pressure: Pathophysiology and therapeutic implications. Vasc. Pharmacol..

[60] Niu L.G., Zhang M.S., Liu Y., Xue W.X., Liu D.B., Zhang J., Liang Y.Q. (2008). Vasorelaxant effect of taurine is diminished by tetraethylammonium in rat isolated arteries. Eur. J. Pharmacol..

[61] Cole W.C., Clément-Chomienne O., Aiello E.A. (1996). Regulation of 4-aminopyridine-sensitive.; delayed rectifier K^+^ channels in vascular smooth muscle by phosphorylation. Biochem. Cell Biol..

[62] Tep-areenan P., Kendall D.A., Randall M.D. (2003). Mechanisms of vasorelaxation to testosterone in the rat aorta. Eur. J. Pharmacol..

